# Low Stress Mechanical Properties of Plasma-Treated Cotton Fabric Subjected to Zinc Oxide-Anti-Microbial Treatment

**DOI:** 10.3390/ma6010314

**Published:** 2013-01-22

**Authors:** Chi-Wai Kan, Yin-Ling Lam

**Affiliations:** Institute of Textiles and Clothing, The Hong Kong Polytechnic University, Hung Hom, Kowloon, Hong Kong, China; E-Mail: astroboyling@yahoo.com

**Keywords:** antimicrobial, cotton, zinc oxide, plasma treatment, low stress mechanical properties

## Abstract

Cotton fabrics are highly popular because of their excellent properties such as regeneration, bio-degradation, softness, affinity to skin and hygroscopic properties. When in contact with the human body, cotton fabrics offer an ideal environment for microbial growth due to their ability to retain oxygen, moisture and warmth, as well as nutrients from spillages and body sweat. Therefore, an anti-microbial coating formulation (Microfresh and Microban together with zinc oxide as catalyst) was developed for cotton fabrics to improve treatment effectiveness. In addition, plasma technology was employed in the study which roughened the surface of the materials, improving the loading of zinc oxides on the surface. In this study, the low stress mechanical properties of plasma pre-treated and/or anti-microbial-treated cotton fabric were studied. The overall results show that the specimens had improved bending properties when zinc oxides were added in the anti-microbial coating recipe. Also, without plasma pre-treatment, anti-microbial-treatment of cotton fabric had a positive effect only on tensile resilience, shear stress at 0.5° and compressional energy, while plasma-treated specimens had better overall tensile properties even after anti-microbial treatment.

## 1. Introduction

Many apparel products are made of cotton because their porous hydrophilic structure offers comfort in wear [1]. However, the ability of cotton textiles to retain moisture, oxygen and nutrients results in an ideal medium to accommodate microbes. These microbes have many negative effects such as generation of unpleasant odors, stains, decolorisation of the material and a decrease in fabric mechanical strength [2,3]. Hence, the quest for efficient, non-toxic, durable and cost-effective antimicrobial finishing of textile materials is becoming increasingly intense, resulting in progressive expansion of the production of anti-microbial textile materials [2,4]. In previous researches [5,6], cotton fabric samples were coated with Microfresh Liquid Formulation 9200-200 and Microban Liquid Formulation R10800-0 (MF-MB anti-microbial formulation) to impart anti-microbial properties. It was proved that the addition of zinc oxide (ZnO) as a catalyst can further enhance the anti-microbial properties. Since the application of the anti-microbial finishing depends much on the surface properties of the material, the modification of the material surface would enhance the functional effect. Therefore plasma treatment appears promising as a good surface modification method for textile materials [7]. It is believed that after plasma treatment, the material surface reactivity would be improved and hence the anti-microbial finishing process would be subsequently enhanced [7]. Also the hydrophilic nature of the carbonyl groups in oxygen plasma pre-treated specimens would increase anti-microbial activity, after treating them with MF-MB. However, there are great difficulties involved in meeting fabric handle requirements of cotton fabrics when treating fabrics with acidic chemical agents. The Kawabata Evaluation System for Fabrics (KES-F) is used in this study for determining low stress mechanical properties in terms of tensile, shearing, bending, compression and surface properties, which are commonly used for expressing fabric handle in textile and apparel sectors, to evaluate effects of plasma pre-treatment and anti-microbial treatment of cotton fabrics. This paper may provide useful information for researchers in the fields of textiles, healthcare and plasma.

## 2. Experimental Section

### 2.1. Material

Semi-bleached plain weave cotton fabric (58 ends/cm, yarn count 40 tex, in warp; 58 picks/cm, yarn count 38 tex, in weft; fabric weight 175 g/m^2^), of size 30 cm × 30 cm was used. The antimicrobial finishing agent and binder used was a halogenated phenoxy compound (Microfresh Liquid Formulation 9200-200, MF) and a polyurethane dispersion (Microban Liquid Formulation R10800-0, MB), supplied by DyStar Textilfarben GmbH & Co. Catalysts used were zinc oxide (ZnO, 2 μm diameter) and nano-zinc oxide (nano-ZnO, 100nm diameter) obtained from Fluka Chemical Corp. and Sigma-Aldrich Co., respectively, both having purity of 99%. All other chemicals used in the study were reagent grade.

### 2.2. Plasma Pre-Treatment

Plasma pre-treatment of cotton fabric was carried out by an atmospheric pressure plasma jet apparatus, Atomflo 400 Plasma controller integrated with a robot, manufactured by Surfx Technologies. The cotton fabric was moved automatically at a speed of 10 mm/s. The machine produced a stable discharge at atmospheric pressure with radio frequency and 120 W output power. The treatment was carried out using a rectangular nozzle which covered an active area of 50.8 mm × 1 mm, mounted vertically, above the cotton fabric. Helium (30 L/min) and oxygen (0.2 L/min) were used as carrier and reactive gases, respectively. Plasma pre-treatment of cotton fabric was conducted at 3 mm jet-to-substrate distance.

### 2.3. Two-Bath Pad-Dry-Cure Antimicrobial Treatment

Plasma pre-treated cotton fabric samples were treated with antimicrobial agents of different compositions (Table 1). A two-bath method was used for the treatments. In the first bath, fabrics were dipped and padded with antimicrobial agents (MF-MB) until a wet pick up of 80% was achieved at 25 °C. The fabrics were then dried at 140 °C for 5 min. In the second bath, dipping and padding processes (80% wet pick up) were performed with ZnO or nano-ZnO solution dispersed in 10% Matexil DN-VL (dispersing agent). Subsequently, padded fabrics were dried at 140 °C for 5 min. Finally, the fabrics were conditioned at 21 ± 1 °C and 65% ± 5% RH for 24 h, prior to any further treatment.

**Table 1:** Antimicrobial treatment conditions.

Sample Symbol	Plasma pre-treatment	Concentrations of reagents			
		Microfresh	Microban	Zinc Oxide	Nano-Zinc Oxide
M1	No	0.25%	0.5%	–	–
M2	No	0.25%	0.5%	0.2%	–
M3	No	0.25%	0.5%	–	0.2%
PM1	Yes	0.25%	0.5%	–	–
PM2	Yes	0.25%	0.5%	0.2%	–
PM3	Yes	0.25%	0.5%	–	0.2%

* Concentration percentage measured based on weight of volume.

### 2.4. Scanning Electron Microscopy (SEM)

The surface morphology of cotton fibers was examined by the JEOL JSM-6490 Scanning Electron Microscope, with an accelerating voltage of 20kV and a current of 10 μA at a high magnification between 2000×–3000×. 

### 2.5. Energy Dispersive X-ray (EDX) Analysis

EDX was used to collect the elemental information of cotton specimens. The measurement was conducted by the JEOL JSM-6490 Scanning Electron Microscope equipped with a cathode and magnetic lenses, to create and focus a beam of electrons for elemental analysis. A detector was used to convert the X-ray energy into voltage signals that were sent to a pulse processor which measured and transmitted the signals to an analyzer for data display and analysis. 

### 2.6. Fourier Transform Infrared Spectroscopy

Chemical composition of the surface of cotton was studied with the Fourier Transform Infrared spectrophotometer (Perkin Elmer Spectrum 100) with the scanning range between 4000 cm^−1^ and 700 cm^−1^ and a resolution of 4 nm^−1^, using attenuated total reflection. The average number of scans was 256; and the area of the relevant signal in the zero-order derivative spectrum was measured.

### 2.7. Kawabata Evaluation System for Fabrics (KES-F)

The Kawabata Evaluation System for Fabric (KES-F) was used for measuring the low-stress mechanical properties of the fabric samples which include the tensile, shearing, bending, compression and surface properties. The parameters obtained for these hysteresis curves are defined in Table 2. Four fabric specimens (200 mm × 200 mm) were tested and the data was collected and averaged statistically with confidence level of 95%. All specimens were conditioned under standard conditions for 24 h prior to all measurements.

**Table 2:** Low stress mechanical properties obtained by the Kawabata Evaluation System for Fabric (KES-F) system.

Properties	Symbol	Definition	Characteristics	Unit
Tensile energy/tensile work	WT	Energy used for extending fabric to 500 gf/cm width.	WT refers to the ability of a fabric to withstand external stress during extension. A fabric with good tensile strength and toughness will have a large value of WT.	gf cm/cm^2^
Tensile resilience	RT	Percentage energy recovery from tensile deformation.	The reduced fabric RT value implies that the fabric becomes difficult to restore to its original shape after releasing the applied tensile stress.	%
Extensibility	EMT	Percentage extension at the maximum applied load of 500 gf/cm specimen width.	EMT has a good correlation with fabric handle. The greater the value of EMT, the larger the elongation of the fabric under a known applied stress.	%
Shear stiffness/shear rigidity	G	Average slope of the linear regions of the shear hysteresis curve to ±2.5° shear angle.	G refers to the ability of a fabric to resist shear stress which is the ease with which the fibers slide against each other. Lower values indicate less resistance to shearing corresponding to a softer material having better drape.	gf/cm degree
Shear stress at 0.5°	2HG	Average width of the shear hysteresis loop at ±0.5° shear angle.	2HG is the ability of a fabric to recover after applying the shear stress value of 0.5° shear angle. The greater the value of shear stress, the worse the recovery ability of the fabric and the stiffer the fabric.	gf/cm
Shear stress at 5°	2HG5	Average width of the shear hysteresis loop at ±5° shear angle.	2HG5 is the ability of a fabric to recover after applying a shear stress value of 5° shear angle. The greater the value of shear stress, the worse the recovery ability and stiffness of the fabric.	gf/cm
Bending rigidity	B	Average slope of the linear regions of the bending hysteresis curve to 1.5 cm^−1.^	B is the ability of a fabric to resist the bending moment, which is related to the quality of stiffness when a fabric is handled. A higher B value indicates greater resistance to bending.	gf cm^2^/cm
Bending moment	2HB	Average width of the bending hysteresis loop at 0.5cm^−1^ curvature.	2HB refers to the recovery ability of a fabric after being bent. It is measured as a specimen is bent through a range of curvatures from 2.5 cm^−1^ to −2.5 cm^−1^. The smaller the value of 2HB, the better the bending recovery of the fabric will be.	gf cm/cm
Compressional linearity	LC	Linearity of compression-thickness curve.	LC determines the compressibility along with the change in fabric thickness after treatment. High value of LC indicates a fluffy fabric with high compressibility.	–
Compressional energy	WC	Energy used for compressing fabric under 50 gf/cm^2^.	The WC value represents a fluffy feeling of the fabric. The higher the value of WC, the higher the compressibility of the fabric.	gf cm/cm^2^
Compressional resilience	RC	Percentage energy recovery from lateral compression deformation.	RC indicates the recoverability of the fabric after the compression force is removed. A higher value indicates better recovery ability from compression.	%
Fabric thickness at 0.5 gf/cm^2^ pressure	*T*_o_	Fabric thickness at 0.5 gf/cm^2^ pressure.	T_O_ measures the surface thickness at a pressure of 0.5 gf/cm^2^.	mm
Fabric thickness at 50 gf/cm^2^ pressure	*T*_m_	Fabric thickness at 50 gf/cm^2^ pressure.	T_O_ measures the intrinsic thickness at a pressure of 50 gf/cm^2^.	mm
Coefficient of friction	MIU	Coefficient of friction between the fabric surface and a standard contactor.	MIU represents the fabric smoothness, roughness and crispness. The value demonstrates the ratio of the force required to slide the surfaces to the force perpendicular to the surfaces. The higher the value of MIU, the greater the friction of the fabric.	–
Geometrical roughness	SMD	Variation in surface geometry of the fabric.	SMD refers to the fabric surface evenness. The lower the SMD value, the more even the fabric surface.	μm

## 3. Results and Discussion

### 3.1. Surface Analysis

**Figure 1:**
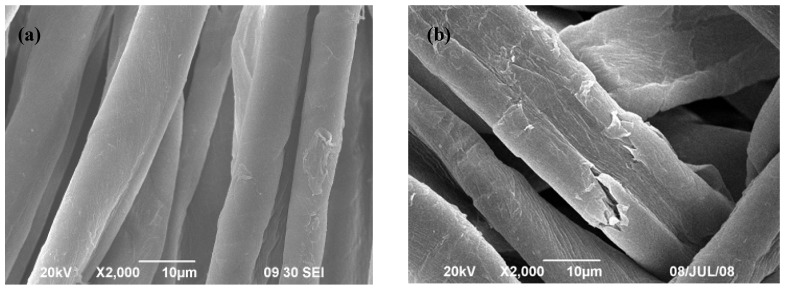
(**a**) Untreated cotton fabric; (**b**) Plasma pre-treated cotton fabric.

Figure 1a,b depict SEM images of untreated and plasma pre-treated fabric respectively. As seen in previous research [6], plasma treatment increased the roughness and the area of the fiber surface compared with the control fabric. Morphological changes induced by plasma treatment can be attributed to fiber etching, which occurs as a consequence of bombardment of the fiber surface by energetic and reactive particles generated by the plasma.

**Figure 2:**
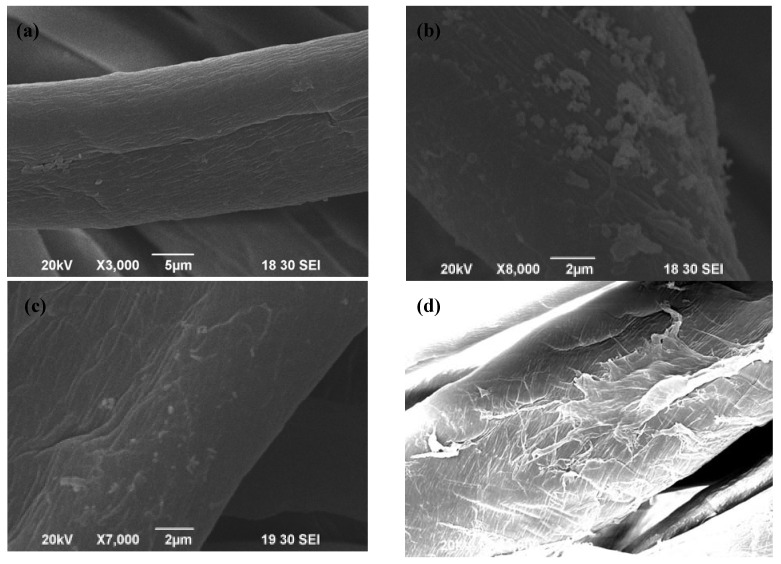

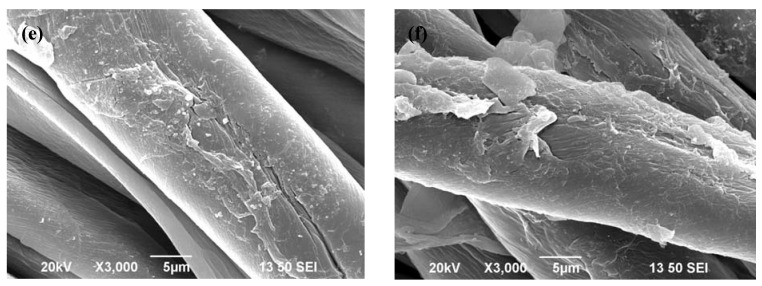
Sample without plasma pre-treatment: (**a**) M1; (**b**) M2 [5]; (**c**) M3 [5]; and sample with plasma pre-treatment: (**d**) PM1 [6]; (**e**) PM2 [6]; (**f**) PM3 [6].

Figure 2a shows the SEM image of a cotton sample treated with 0.25% MF and 0.5% MB. When compared with the control fabric (Figure 1a), the morphological structure of MF-MB-treated specimen showed a rougher and more wrinkled fiber surface. The deposition of finishing agent on the fibers may damage the surface due to the slight acidity of the reagents, *i.e.*, pH 5 as measured. The results indicate that crosslinking may play an important role in coating the cotton surface with anti-microbial agents due to the formation of chemical bonding. 

In addition, Figure 2b,c illustrate the SEM images of the cotton specimen treated with 0.25% MF and 0.5% MB in the presence of 0.2% ZnO and nano-ZnO respectively. Figure 2b,c show that the ZnO and nano-ZnO particles of irregular shape attached to the cotton fabric were agglomerated. The agglomeration of ZnO and nano-ZnO particles of similar size is unevenly distributed on the fiber surface and between the fibers. The ZnO and nano-ZnO agglomerated particles with their diameters in the range of 0.35–1.50 μm and 0.05–1.25 μm respectively were observed. It is believed that the agglomeration of particles was mainly due to the surface attraction between small particles. 

In the case of combined plasma pretreatment and antimicrobial finishing, Figure 2d illustrates that the morphological structure of the plasma-MF-MB treated specimen became rougher and more wrinkled when compared with the MF-MB treated specimen due to the damage caused by the slightly acidic chemical agents, *i.e.*, pH 5 as measured. Furthermore, Figure 2e,f show the agglomeration of irregular-shaped particles of zinc oxide on the fiber surface and between the fibers. In addition, the ZnO and nano-ZnO agglomerated particles with diameters in the range of 0.10–1.00 μm and 0.05–0.30 μm respectively are distributed more evenly than the ones without plasma pretreatment.

EDX was used to collect the elemental information of the anti-microbial and plasma-treated cotton fabrics. The atomic percentage of C, O, Zn and Cl that appeared in the anti-microbial treated fabrics is shown in Table 3. 

**Table 3:** The atomic percentage of different elements present in the anti-microbial treated cotton fabrics with or without plasma pre-treatment.

Sample Symbol	C (%)	O (%)	O/C	Zn (%)	Cl (%)
Control	52.43	47.57	0.90	–	–
M1	51.44	47.81	0.93	–	0.75
M2	50.68	48.30	0.95	0.19	0.83
M3	50.12	48.88	0.97	0.11	0.89
Plasma pre-treatment (PM0)	51.04	48.96	0.96	–	–
PM1	49.85	49.21	0.99	–	0.94
PM2	49.29	49.43	1.00	0.03	1.25
PM3	48.55	50.08	1.03	0.01	1.36

Table 3 shows that the C content dropped by 1% when the fabric was treated with anti-microbial agent (control fabric). The C content dropped by 1.8% and 2.3% when the fabric was treated with anti-microbial agent in the presence of zinc oxide and nano-zinc oxide respectively. In addition, the EDX results also indicated that small amounts of Zn (from zinc oxides) and Cl (from the anti-microbial agent) were present in the fiber after treatment. Atomic percentages of C, O, Zn and Cl that appeared in the plasma pre-treated fabrics are also shown in Table 3. Due to the chemical effects of plasma species, new functional groups were created causing various changes in the surface composition [6]. The EDX analysis revealed the changed quantitative surface composition after the plasma treatment. Table 3 also shows that the C atomic percentage decreased while the oxygen atomic percentage increased. The removal of fiber surface material was related to the drop of C content. The O/C ratio of the plasma pre-treated fabric with or without ant-microbial treatment increased significantly. This was mainly attributed to the incorporation of specific oxygen functional groups formed by the plasma pre-treatment. The increase in O content led to an improvement of both hydrophilicity and wickability. 

**Figure 3:**
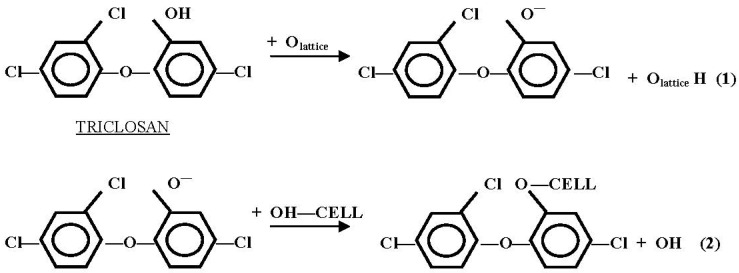
Catalytic reaction between cellulose and triclosan [5,9].

The MF-MB formulation containing triclosan, *i.e.*, 2,4,4'-trichloro-2'-hydroxydiphenyl ether shown in Figure 3, as the active anti-microbial agent was applied at the finishing stage or incorporated into the fiber during extrusion. Triclosan is very effective against a broad range of microorganisms including antibiotic-resistant bacteria [8]. It connects the terminal hydroxyl group in each molecule to cellulose. Since the bonding is relatively weak [8], triclosan has been applied to cellulose fibers in combination with a binder to enhance the washing durability of the anti-microbial coating. When the zinc oxides were added in the treatment, the triclosan molecules were catalysed to provide an alternative reaction pathway for the reaction product. It was proposed that the hydroxyl group of the anti-microbial agent could dissociate at the ZnO surface to form a hydroxyl molecule with a surface atom O_lattice_ as shown in Equation (1) of Figure 3 [9]. Therefore, the presence of ZnO catalyst played an important role in the anti-microbial finish by effectively crosslinking the cellulose and triclosan as shown in Equation (2) of Figure 3.

**Figure 4:**
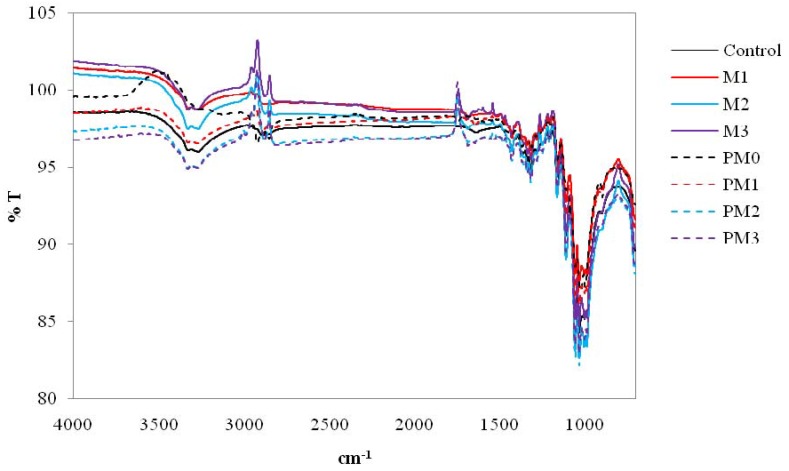
FTIR spectra of anti-microbial treated cotton specimen at 4000 to 700 cm^−1^.

FTIR was used to characterise the bonding present in the anti-microbial treated cotton fabric in order to confirm the reactions presented in Figure 3. The FTIR spectra of the anti-microbial treated fabrics in the presence or absence of zinc oxide catalyst are presented in Figure 4. With regard to M1 specimen, it was observed that there were strong hydroxyl stretching bands at 3420–3250 cm^−1^ representing the presence of triclosan on the fabrics [10]. However, the terminal hydroxyl group of the triclosan molecule might react with cellulose. Hence, there was no new characteristics peak formed as shown in Figure 4. 

**Figure 5:**
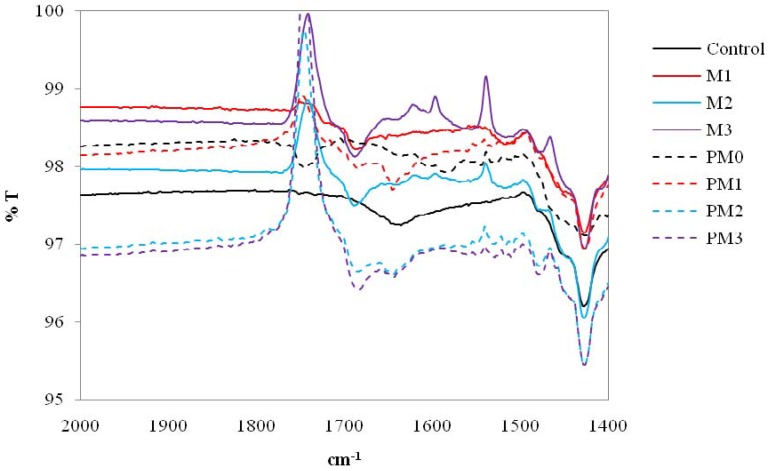
FTIR spectra of anti-microbial treated cotton specimen at 2000 to 1400 cm^−1^.

**Figure 6:**
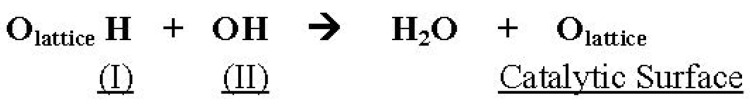
Chain reaction from the catalytic reaction between cellulose and triclosan.

On the other hand, Figure 5 (FTIR spectra of cotton specimens focused at 2000–1400 cm^−1^) shows two medium sharp bands at 1670 and 1510 cm^−1^ respectively which are due to the presence of the benzene ring of aromatic compounds [10]. Therefore, it was confirmed that the anti-microbial agent applied was successfully bonded to the cotton fabrics. In addition, the FTIR spectra as shown in Figure 4 of the zinc oxide-coated cotton fabric, *i.e.*, M2 and M3 specimens, did not reveal any new peak when compared with the M1 specimen, meaning that no chemical bond was formed on the surface of cotton fabrics with zinc oxide [11,12]. The results further confirmed that the zinc oxide used in the anti-microbial finishing could act as a catalyst. Furthermore, FTIR of the spectra of M2 and M3 specimens contained some signals near the baseline. These signals were usually associated with the absorbance of water and carbon dioxide that were cancelled correctly by the background spectrum. Nevertheless, the presence of water absorption might be due to the reaction between two hydroxyl groups formed (molecule (I) formed in Equation (1) (Figure 3) and molecule (II) formed in Equation (2) (Figure 3)) when the O_lattice_ recovered to zinc oxide, as presented in Figure 6 [5,6]. 

From Figure 5, it was found that the characteristic bands related to the benzene rings at 1670 and 1510 cm^−1^ were also observed in the PM1 specimen. In general, FTIR can be used for quantitative analysis because the strength of the absorption is proportional to the concentration. It was confirmed that the PM1 specimen contained more antimicrobial agent than that of the M1 specimen. Furthermore, Figure 5 also shows the presence of weak characteristics peaks at 1540 cm^−^^1^ and at 1660 cm^−1^ due to plasma pre-treatment. COO^−^ stretching vibrations and C=O vibration groups were still observed even after anti-microbial treatment [5,6].

### 3.2. Low Stress Mechanical Properties

There are great difficulties involved in meeting fabric handle requirements of cotton fabrics when treating fabrics with acidic chemical reagents. Sensory tests conducted by KES-F instrumental measurements determine the fabric handle, which is a critical physical property for consumers when making purchasing decisions [13]. The KES-F system comprises five specialised instruments for testing tensile strength, shearing strength, bending, compression and surface friction and variation. The results of testing of these properties are presented in Table 4.

#### 3.2.1. Tensile Properties

The study of tensile properties of fabrics, including tensile energy (WT), tensile resilience (RT) and extensibility (EMT) is important because apparel usually undergoes small extension and relaxation during wear. WT represents the energy required for extending a fabric of a specific length without damaging it, which indicates the toughness of the fabric and reflects the mobility of the garment under deformation [14,15,16]. On the other hand, EMT is determined as the percentage extension at the maximum applied load of 500 gf/cm of the specimen’s width, which has a good correlation with fabric handle. In general, WT and EMT are highly correlated and high WT and EMT values imply better tensile strength. As shown in Table 4, WT and EMT values of the control specimen are 15.10 gf cm/cm^2^ and 9.14%, respectively, and a significant drop was observed after anti-microbial treatment. The loss of strength is mainly due to the destructive squeezing and padding and high temperature drying process. The M1 specimen is further tendered at low pH treatment, *i.e.*, pH 5, resulting in poorer tensile strength and toughness. In addition, WT and EMT values of M2 and M3 specimens drop clearly when compared to the control specimen. This is because a two-bath pad-dry method was used for treatment of the cotton fabric, implying that the fabrics twice underwent padding and drying processes. Therefore, the strength loss is mainly attributed to the fabric having twice undergone padding and drying processes. Furthermore, the results show that the change in tensile strength is not related to the type of zinc oxide applied.

In addition, plasma treatment etched the fabric surface and the roughening effect created a larger contact area of fibers and yarns [17,18,19,20]. Table 4 shows that WT and EMT values of the fabric, compared with the control fabric, were slightly enhanced (7.3% and 4.6% respectively) by the plasma treatment. The plasma treatment also enhances inter-yarn and inter-fiber friction because of the etching effect [17,18,19,20] and hence, the increased WT value was probably due to the greater cohesive force developed during extension. On the other hand, the increased EMT value might be due to the rise of interaction forces between fibers and yarns, leading to a slight reduction in the relative movement of fibers and yarns during extension. When the plasma pre-treated specimen was subjected to the MF-MB treatment (PM1 specimen), WT and EMT values dropped but were still higher than in the M1 specimen. The results confirmed that the plasma pre-treatment can help minimize side effects of anti-microbial treatment. Similarly, the trends shown in WT and EMT values of PM2 and PM3 specimens are similar to M2 and M3 due to the plasma pre-treatment. 

In general, fabrics with high RT values have better recovery after tensile deformation, *i.e.* better tensile strength. Table 4 shows that the control sample had 38.59% RT, which was the lowest among other specimens. MB functioned as a binder to form a linkage between the treated cotton fibers and the crosslinked structure imparted elasticity to the fabric. The results showed that both WT and EMT values of the M1 specimen decrease but the RT value increases which contributes to the reduction of tensile strength and a slight improvement of elastic properties. In addition, M2 and M3 specimens are coated with zinc oxide particles on the fabric surface and filled between the fibers, which might have resulted in resistance to the tensile stress as the test specimens recovered to their original shape easily in comparison with the control fabric. Nevertheless, the results show that the change in RT value is not related to the type of zinc oxide applied. On the other hand, in the case of MF-MB-treated cotton fabrics coated with zinc oxides in the second bath (M2 and M3 specimens), there is a decrease in RT value when compared with the M1 specimen. Although the zinc oxides, used as catalysts in the MF-MB treatment, could improve the catalytic reaction of MF-MB leading to enhancement of RT, the two padding and drying processes resulted in formation of tangled fibrils during wet processing. Apart from this, wet treatment swells the fibers, resulting in fabric shrinkage. In general, the wet process breaks the hydrogen bonds formed between fibers when in the swollen state and allows the yarn to collapse inwardly, thereby reducing yarn diameter and facilitating shrinkage [21,22]. Hence, the shrinkage effect and the formation of tangled fibers negated the benefits obtained from the catalytic reactions; it became difficult for the M2 and M3 specimens to recover to their original shape after release of the applied tensile stress, *i.e.*, the drop of RT value.

**Table 4:** Low stress mechanical properties of cotton specimens subjected to anti-microbial treatment. (See Table 2 for definitions of properties).

Sample Symbol	WT (gf cm/cm^2^)	RT (%)	EMT (%)	G (gf/cm degree)	2HG (gf/cm)	2HG5 (gf/cm)	B (gf cm^2^/cm)	2HB (gf cm/cm)	*T*_m_ (mm)	*T*_o_ (mm)	LC	WC (gf cm/cm^2^)	RC (%)	MIU	SMD (μm)
Control	15.10	38.59	9.14	2.81	5.38	7.84	0.103	0.113	0.637	0.975	0.36	0.30	36.40	0.20	5.48
M1	12.95	46.07	7.60	3.04	3.66	8.12	0.107	0.114	0.607	1.027	0.34	0.40	36.23	0.22	6.12
M2	12.59	44.76	7.39	3.13	3.86	8.21	0.101	0.099	0.581	0.978	0.33	0.35	34.86	0.25	6.98
M3	12.66	44.43	7.39	3.08	4.01	8.35	0.102	0.099	0.579	0.976	0.34	0.34	32.03	0.23	7.09
Plasma-treated only	16.20	42.35	9.56	2.91	6.78	9.41	0.114	0.146	0.524	0.841	0.30	0.24	35.37	0.24	5.95
PM1	13.14	47.08	7.73	3.07	3.97	8.10	0.109	0.119	0.604	1.045	0.33	0.40	34.41	0.22	6.51
PM2	12.64	44.47	7.49	3.09	4.44	8.65	0.102	0.113	0.586	1.034	0.32	0.37	29.73	0.22	6.95
PM3	12.68	44.54	7.45	3.12	4.46	8.42	0.104	0.113	0.579	1.050	0.32	0.38	31.44	0.21	6.96

Furthermore, there is an obvious increase in RT value (9.7%) compared with the control fabric, after the plasma treatment (Table 4). This is probably due to removal of fuzz and tangled fibrils, which imparted to the fabric a greater ability to recover from stretching. When plasma pre-treated fabrics were subjected to anti-microbial treatment, the PM1 specimen showed the highest RT values because the fuzzy fibrils on the fabric surface are removed while at the same time, plasma pre-treatment enhanced the absorption of MF-MB, resulting in a better crosslinked structure having a greater ability to recover from stretching. In addition, zinc oxides coated on the PM2 and PM3 specimens could fill on or between the fibers effectively, due to the roughening effects of plasma pre-treatment. Therefore, the PM2 and PM3 specimens can resist the tensile stress and recover to their original shape easier in comparison with the M2 and M3 specimens. The results also show that the change in RT values is not related to the type of zinc oxides applied. Moreover, RT values of the PM2 and PM3 specimens are slightly reduced when compared with the PM1 specimen. When the plasma pre-treated specimen was subjected to two padding and drying processes, surface fibrils re-developed during the wet padding process. Hence, the reduction in RT value is due to the second padding with zinc oxide solution followed by drying. Besides, the RT values of the PM2 and PM3 specimens and the M2 and M3 specimens are similar, implying that the shrinkage effect and formation of tangled fibers negated the benefits obtained from catalytic reactions and plasma pre-treatment.

#### 3.2.2. Shearing Properties

The study of shearing properties of a fabric includes shear stiffness (G) and shear stress at 0.5° (2HG) and 5° (2HG5). Shear is a significant determinant of handle and drape of fabrics.A fabric with low G, 2HG and 2HG5 values shows superior shearing properties. Table 4 indicates that the G value, defined as the ease with which fibers slide against each other, of the control specimen, is the lowest. For the M1, M2 and M3 specimens, there is a slight increase in G values compared with the control fabric. Morphological studies discussed in existing literature show that zinc oxide particles are attached to the cotton fabric during the padding process but they are unevenly distributed on the fiber surface with varying sizes of particles [5,6]. The enhancement in G values could possibly be attributed to the roughened fabric surface with a certain degree of agglomeration of particles. Moreover, Table 4 demonstrates that enhancement of G values is more obvious when ZnO (M2 and M3 specimens) particles are added in the treatment solution. These relatively larger particles on the fabric surfaces could cause more severe rigidity, while the nano-ZnO present on the surface in the M3 specimen could be filled between the fibers with less effect on shear rigidity of the fabric. In addition, there is a remarkable increase in G values of the M1 specimen implying a stiffer hand feel. It is evident that the pH value of the reaction medium used for the M1 specimen was pH 5, which might have led to higher stiffness. Also, the MB binder can lead to formation of brittle polymer layers although mobility of cellulose macromolecules is limited. With the combined effects of acidic treatment and roughness of zinc oxides, the M2 and M3 specimens demonstrated significant enhancement in G values when compared with the control fabric.

An increase in G value is observed in plasma-treated specimens when compared with the control fabric indicating enhancement of subjective stiffness of the fabric. This is primarily dependent on yarn interaction, *i.e.*, an increase in yarn interaction normally increases shear rigidity, and is perhaps related to the smoothing effect [16,23]. The results in Table 4 indicate the greatly increased inter-yarn friction in plasma-treated fabrics as well as the increase in the number of fiber contacts at yarn crossover points generating inter-yarn pressure. Moreover, Table 4 shows that the rigidity effect is even worse in plasma pre-treated cotton specimens with zinc oxide or anti-microbial treatment, *i.e.*, a further increase in G values. This phenomenon is attributed to the etching effect on the fabric surface caused by plasma pre-treatment which roughened it. As a result, the etched fabric provides a new pathway for the finishing agent to enter into the fibers, leading to a more effective after-treatment. In addition, the increased wettability of cotton fibers might also have facilitated absorption of chemicals, leading to enhanced G values.

In addition to the G value, both 2HG and 2HG5 determine shearing properties of fabrics, *i.e.*, the greater the value of the shear stress, the worse is the recovery ability of the fabric and the stiffer the fabric. As shown in Table 4, 2HG and 2HG5 values of the control specimen are 5.38 gf/cm and 7.84 gf/cm, respectively. After MF-MB treatment, the 2HG value of the M1 specimen drops significantly. This is attributed to the glue-like MB film on the fabric surface imparting a slightly elastic property to the fabric which helps recovery when subjected to small distortion. However, the poor fabric recovery ability after applying shearing stress is reflected by the shear stress value from 0.5° to 5° shear angle. Table 4 indicates that the 2HG5 value of the M1 specimen is slightly increased when compared with the control fabric. The poor recovery ability is probably due to the limited extension and recovery of the glue-like MB film on the fabric at a larger shear angle. 

The 2HG and 2HG5 values of the M2 and M3 specimens increase in comparison with the M1 specimen. In general, shear hysteresis 2HG and 2HG5 represents the energy that the fabric loses during shear deformation; the energy loss is caused primarily by friction occurring at points where warp and weft yarns cross each other [24]. Hence, the increase in 2HG and 2HG5 values is mainly due to the presence of zinc oxide particles between the fibers or adherance to the fibers that increases the friction and obstructs the fabrics from reverting to their original state after shear stress was applied at both 0.5° and 5°. Moreover, for the M2 and M3 specimens, the 2HG and 2HG5 values are higher than the control specimen. The results show deterioration in the ability of the M2 and M2 specimens to recover after being subjected to shear stress at 0.5° and 5° shear angle, which may have been accentuated by the presence of zinc oxides. However, the results show that the change in 2HG and 2HG5 values is irrespective of the type of zinc oxides applied.

Furthermore, Table 4 indicates that the 2HG and 2HG5 values of plasma-treated cotton fabrics are increased when compared with the control specimen. This can probably be attributed to the fact that inter-yarn friction of the plasma-treated specimen is greatly increased, besides the increased number of fiber contacts at the yarn crossover points and inter-yarn pressure. The increased friction and contact points resulted in enhancement of the 2HG and 2HG5 values. As shown in Table 4, the results indicate that the 2HG and 2HG5 values for the PM1 to PM2 specimens are slightly increased when compared with M1 and M2, implying poor recovery ability. The poor fabric recovering ability is attributed to both the plasma etching effect and the presence of zinc oxides. 

#### 3.2.3. Bending Properties

The bending properties of a fabric include bending rigidity (B) and bending moment (2HB), which depends on the bending resistance and the friction between fibers and yarns, as well as the fabric structure [25,26]. B is the ability of a fabric to resist the bending moment, which is related to the quality of stiffness when a fabric is handled. On the other hand, 2HB describes the bending behavior of a fabric subjected to an external load applied perpendicular at the curvatures from 2.5 cm^−1^ to −2.5 cm^−1^ to a longitudinal axis of the fabric. Generally speaking, fabrics with low B and 2HB values have better bending properties. 

Table 4 shows that the M2 and M3 specimens have decreased B values when compared to the control fabric. It is worth noting that fabrics undergoing wet treatment resulted in lower bending resistance, probably due to degradation emphasized by the mechanical damage caused by the padding process. In addition, there was a slight increase in the B value of the the M1 specimen when compared with the control fabric. It was evident that the presence of MB binders on the fabrics may enhance fabric stiffness. Besides, pH5 reaction medium may lead to the formation of brittle and tendered polymer layers, making the test specimens stiffer. When MF-MB specimens are treated with different zinc oxides, the B values of the M2 and M3 specimens are decreased (compared with the M1 specimen), as shown in Table 4. This is attributed to the fact that the unattached acidic residues on the specimens were washed and diluted in the second bath of zinc oxides during padding. A softer and smoother material is thus obtained.

In addition, Table 4 indicates that the B value of the control specimen changed from the original 0.103 gf cm^2^/cm to 0.114 gf cm^2^/cm after plasma treatment. This is primarily attributed to enhancement of yarn interaction, leading to an increase in inter-yarn friction and number of fiber contacts at yarn crossover points and inter-yarn pressure in plasma-treated fabrics, *i.e.*, increase in the bending rigidity. Similarly, Table 4 demonstrates that the trend shown in B values of PM2 and PM3 specimens are similar to M2 and M2, but with slight enhancement in B values contributed by the plasma pre-treatment.

The 2HB value of the control specimen is 0.113 gf cm/cm which is slightly increased after anti-microbial treatment. It is assumed that the binder imparted an elastic property to the fabrics. However, the low pH reaction medium weakened the fabrics, leading to poor recovering ability upon bending at curvatures from 2.5 cm^−1^ to −2.5 cm^−1^. On the other hand, the 2HB values of the M2 and M3 specimens decreased which is mainly due to removal of excessive acids when the specimens were padded in the second bath of zinc oxides. In addition, Table 4 shows that the M2 and M3 specimens have an obvious decrease in 2HB values when compared to the control fabric. It is of note that the fabric undergoing wet treatment could have resulted in fuzzy or tangled surface fibrils and the fibrils would have pulled outwards when the specimens were bent. As a result, the crimp in the fiber provides an elastic characteristic to restore the fibers to their original shape. Therefore, the overall 2HB values decrease proportionally after zinc oxide treatment. Furthermore, the results show that the changes in 2HB values are not related to the type of zinc oxides applied. In addition, Table 4 indicates that the 2HB value is increased remarkably after plasma treatment. This is primarily attributed to enhancement of inter-yarn friction and the number of fiber contacts at yarn crossover points in plasma-treated fabrics. Similarly, Table 4 shows that the trends in the B values of the PM2 and PM3 specimens are similar to the M2 and M3 specimens but with a lower percentage reduction in 2HB values. This is mainly attributed to removal of surface fibrils by the plasma pre-treatment, minimizing the elastic characteristic provided by the surface fibrils.

#### 3.2.4. Compression Properties

Compression properties of the cotton specimens, such as fabric thickness, at a pressure of 0.5 gf/cm^2^ (*T*_o_) and 50 gf/cm^2^ (*T*_m_), compressional linearity (LC), compressional energy (WC) and compressional resilience (RC) were measured at three distinct points on the specimens. Fabric thickness always changes upon any physical or chemical treatment and is measured as *T*_o_ and *T*_m_, representing the surface and intrinsic thickness, respectively. Table 4 indicates that *T*_o_ and *T*_m_ of the control fabric is 0.975 mm and 0.637 mm, respectively. However, *T*_m_ of the M2 and M3 specimens decreases after anti-microbial treatment; the M2 and M3 specimens are dipped and padded with MF-MB and zinc oxides solution respectively. Both the compression in the padding process and the natural fabric shrinkage in wet conditions, compete with each other, resulting in changing the fabric’s intrinsic thickness. When compared with the control fabric, the results show that there is a 4.7% reduction in T_m_ of M1 specimens, while there is a reduction in T_m_ of M2 and M3 specimens in the range of 8.8%–9.1% respectively. It is believed that the reduction is mainly contributed by the pressure generated between the two padding rollers. Nevertheless, the M1 specimen has a higher *T*_m_ than the M2 and M3 specimens probably due to the deposition of the MF-MB film on the fabric surface. On the other hand, the M2 and M3 specimens have an obvious reduction in fabric *T*_m_. The M2 and M3 specimens are dipped and padded twice, first with anti-microbial agents and then with zinc oxide solutions. It is believed that further reduction in fabric thickness is due to double compression applied to the fabrics during padding. In addition, *T*_o_ of the M1, M2 and M3 specimens is increased when compared with the control fabric. This is probably due to the fact that wet padding process might develop fuzzy surface fibrils randomly. Hence, the sponge-like fibril structure enhances *T*_o_ of the M1, M2 and M3 specimens. 

Moreover, the etching effect of plasma pre-treatment removes the fuzzy fibrils present on the fabric surface resulting in a decrease of *T*_m_ and *T*_o_. Therefore, as shown in Table 4, plasma-treated specimens have *T*_m_ and *T*_o_ of only 0.524mm and 0.841 respectively. However, *T*_m_ and *T*_o_ of PM1 to PM2 are enhanced after anti-microbial or zinc oxide treatment. Specimens undergoing the wet padding process might shrink and create fuzzy fibrils which dominate the etching effect generated from the plasma pre-treatment and the compression effect created by padding, resulting in the formation of a dense fabric with a higher *T*_o_ and *T*_m_.

Apart from fabric thickness, the KES-F instrument also measures LC, WC and RC of anti-microbial-treated and plasma-anti-microbial-treated cotton fabrics. It is suggested that fabrics with good compression properties usually possess higher LC, WC and RC values. In general, WC represents the fluffy feeling of a fabric and hence Table 4 presents a similar trend in *T*_o_ and WC values. The WC value of the control fabric is 0.30 gf cm/cm^2^ and the WC values of M1, M2 and M3 specimens increase when compared with the control fabric, which is probably due to the development of fuzzy surface fibrils during padding and drying. Moreover, the etching effect of plasma treatment could remove the fuzzy fibrils present on the fabric surface, resulting in a 20.0% decrease in WC values when compared with the control fabric. However, the WC values of PM1, PM2 and PM3, after undergoing the wet padding process, are enhanced after anti-microbial or zinc oxide treatment. This was mainly due to the fuzzy fibrils re-development after padding and drying.

On the other hand, the LC determines the compressibility along with the change in fabric thickness. Table 4 shows that the LC value of the control fabric was 0.36. However, after anti-microbial treatment there is a downward tendency in LC values, especially when the specimens are treated twice with the padding process, *i.e.*, M2 and M3 specimens. This is attributed to the fact that the specimens were compressed when squeezed between two rollers, which required higher energy for compressing a dense fabric, leading to poor compressibility. Besides, dipping and padding of M2 and M3 specimens twice created fuzzy fibrils on the fabric surface. It is believed that the fluffy fabric can act as a sponge-like structure with high compressibility. However, the LC values of these specimens are still lower than the control specimen, as the padding process applied twice still compressed the fabrics. Moreover, the plasma surface modification removed fuzzy fibrils on the fabric surface, resulting in LC values lower than the control fabric. Moreover, the effect can be overcome by the post-dipping and post-padding processes as the surface-raising of the cotton fabric during the post-treatment provided a highly compressible structure. The inconsistent decreases in the LC values may be indicative of random development of tangled fibrils after the wet dipping process.

RC refers to the percentage of energy recovery, which measures the percentage of energy recovery from the lateral compression deformation, indicating the recoverability of a fabric after the compression force has been removed. The RC value of the control specimen is 36.40% but it decreases after anti-microbial treatment. Although the binder on the cotton surface could fix the position of the fibers and impart a slight recovering ability after deformation, there is still a slight reduction in the RC values of the M1 specimen. This is probably due to the fact that the M1 specimen is compressed and squeezed between two rollers, leading to a dense fabric structure. It is even worse for the M2 and M3 specimens that are padded twice. Hence, higher energy is required to recover from lateral compression deformation, *i.e.*, a further drop in RC values. Moreover, there is an obvious reduction of recoverability of fabric when only zinc oxides are added to the finishing recipes. In addition, plasma pre-treatment decreases slightly the RC values, implying that compressibility of plasma pre-treated fabrics is lower than for untreated fabrics. It is evident that the fuzzy fibrils, *i.e.* the sponge-like structure, formed on the fabric surface, are removed by plasma pre-treatment. Owing to the absence of the sponge-like structure, recoverability of the fabric decreases slightly after compression. Moreover, the RC values of the PM1, PM2 and PM3 specimens are further reduced accordingly. 

#### 3.2.5. Surface Properties

Surface properties, including the coefficient of friction (MIU) and the geometrical roughness (SMD), of plasma-treated specimens are as shown in Table 4. Generally speaking, fabrics with low MIU and SMD values have better surface properties. There is an increase in MIU and SMD values of the control sample after antimicrobial treatment and plasma pre-treatment. In comparison with the control specimen, the increase in MIU and SMD values of the M1 specimen after the MF-MB treatment is probably due to the attack of acidic chemicals which roughens the fabric surface. Besides, the roughened fabric surface of the MF-MB-treated specimen is also attributed to the development of fuzzy fibrils due to the tendering of fibers after anti-microbial treatment in acidic conditions and the rubbing of fibers during the wet processing. Hence, it is suggested that the fabric surface friction is enhanced and the ratio of the force required to slide the surfaces to the force perpendicular to the surface is increased. In addition, morphological studies in previous researches have showed that zinc oxide particles attached to the cotton fabric are unevenly distributed on the fiber surface with great variation of particle size [5,6]. Results in Table 4 show that fabric roughness is further enhanced after addition of zincs oxide particles on the fabric surface, *i.e.*, an increase in MIU and SMD values for the M2 and M3 specimens. These results might be attributed to a certain degree of agglomeration of zinc oxide particles. The greater the zinc oxide particles present on the fabric surface, the rougher the fabrics would be which is reflected in the MIU and SMD values. 

Moreover, it is obvious that MIU and SMD values increase significantly after plasma treatment. The increased surface roughness is mainly attributed to the etching effect caused by bombardment of plasma on the cotton specimens [22]. When compared with the specimen treated with plasma gas only, MIU and SMD values of plasma pre-treated specimens with different antimicrobial treatments are enhanced accordingly. However, percent enhancements in the MIU and SMD values of M1, M2 and M3 were similar to PM1, PM2 and PM3. As a matter of fact, surface friction of the specimens is determined mainly by the existence of surface fibrils and presence of zinc oxides.

## 4. Conclusions 

In this study, MF-MB-ZnO and MF-MB-nano-ZnO coating formulations were used as effective antimicrobial agents for coating cotton fabric, with or without plasma pretreatment. The study shows that fabric handle might change after plasma pretreatment and/or anti-microbial treatment. The overall results reveal that without plasma pre-treatment, anti-microbial-treated cotton fabrics have only a positive effect on tensile resilience, shear stress at 0.5°, and compressional energy. When zinc oxides were added in the anti-microbial recipe, the specimens had improved bending properties. On the other hand, the plasma-treated specimen had better overall tensile properties after anti-microbial treatment. Hence, it was confirmed that ZnO catalyst in the anti-microbial formulation improves treatment effectiveness and minimizes the side effects of the treatment. In addition, pretreatment of cotton fabrics by plasma was employed to improve the loading of chemical reagents as well as to compensate for the loss of mechanical strength. 
